# Recent Hydroxychloroquine Use Is Not Significantly Associated with Positive PCR Results for SARS-CoV-2: A Nationwide Observational Study in South Korea

**DOI:** 10.3390/v13020329

**Published:** 2021-02-20

**Authors:** Seongman Bae, Byeongzu Ghang, Ye-Jee Kim, Joon Seo Lim, Sung-Cheol Yun, Yong-Gil Kim, Sang-Oh Lee, Sung-Han Kim

**Affiliations:** 1 Asan Medical Center, Department of Infectious Diseases, University of Ulsan College of Medicine, Seoul 05505, Korea; songman.b@gmail.com (S.B.); soleemd@amc.seoul.kr (S.-O.L.); 2 Division of Rheumatology, Department of Internal Medicine, Jeju National University School of Medicine, Jeju National University Hospital, Jeju 63241, Korea; indream81@naver.com; 3 Asan Medical Center, Department of Clinical Epidemiology and Biostatistics, University of Ulsan College of Medicine, Seoul 05505, Korea; kimyejee@amc.seoul.kr (Y.-J.K.); ysch97@amc.seoul.kr (S.-C.Y.); 4 Clinical Research Center, Asan Medical Center, Asan Institute for Life Sciences, University of Ulsan College of Medicine, Seoul 05505, Korea; joonseolim@gmail.com; 5 Asan Medical Center, Division of Rheumatology, Department of Internal Medicine, University of Ulsan College of Medicine, Seoul 05505, Korea; bestmd2000@amc.seoul.kr

**Keywords:** hydroxychloroquine, SARS-CoV-2, COVID-19, prophylaxis

## Abstract

Background: To evaluate the role of hydroxychloroquine (HCQ) as pre-exposure prophylaxis against coronavirus disease 2019 (COVID-19), we investigated the prevalence of positive test results for severe acute respiratory syndrome coronavirus 2 (SARS-CoV-2) testing according to recent HCQ use in patients who had been tested using nationwide health-insurance data of South Korea. Methods: All adults tested for SARS-CoV-2 from 20 January 2020 to 15 May 2020 were identified. HCQ users were defined as patients who had been pretreated with HCQ for at least 30 days until the date of SARS-CoV-2 testing. The prevalence of positive PCR results for SARS-CoV-2 was compared between HCQ users and nonusers. Results: Of a total of 216,686 individuals who had been tested for SARS-CoV-2, 743 (0.3%) were pretreated with HCQ. The prevalence of positive results was not significantly different between HCQ users (2.2%) and nonusers (2.7%; *P* = 0.35), with an odds ratio of 0.79 (95% confidence interval (CI), 0.48–1.30). Propensity score-matched-cohort analysis showed similar results in terms of the prevalence of positive results (2.2% in HCQ users vs. 3.1% in nonusers; *P* = 0.18), with an odds ratio of 0.69 (95% CI, 0.40–1.19). The rate of positive PCR was not significantly different in long-term HCQ users (more than 3 or 6 months) compared with nonusers. Conclusions: In this population-based study, recent exposure to HCQ was not significantly associated with a lower risk of SARS-CoV-2 infection. Our data do not support the use of HCQ as pre-exposure prophylaxis against COVID-19.

## 1. Introduction

In the current pandemic of coronavirus disease 2019 (COVID-19), various drugs are being investigated for possible repurposing to treat against severe acute respiratory syndrome coronavirus 2 (SARS-CoV-2) [1,2,3]. However, no drugs or chemicals have yet proven to be effective in treating or preventing COVID-19 except for remdesivir or a combination of interferon beta-1b, lopinavir–ritonavir, and ribavirin, albeit with limited clinical experience [4,5,6]. 

Hydroxychloroquine (HCQ), an aminoquinoline derivative with decades of clinical experience for treating malaria and rheumatic diseases, has received great attention due to its potential therapeutic or prophylactic effects against SARS-CoV-2 in in vitro studies [7,8,9]. HCQ can increase endosomal pH and interfere with the terminal glycosylation of angiotensin-converting enzyme 2 (ACE2). The fusion of the virus to the host cell can be blocked by elevated endosomal pH, and the binding affinity of the viral spike protein to the host cell receptor, ACE2, can be reduced by the underglycosylation of ACE2 [7,8]. Several preliminary reports and retrospective studies suggested the antiviral effect of HCQ in clinical settings, but other studies including randomized clinical trials did not show significant therapeutic benefits of the drug [10,11,12,13]. In addition, the use of HCQ as postexposure prophylaxis failed to prevent COVID-19 in a clinical trial, indicating the limited or absent role of the drug against SARS-CoV-2 [14]. However, the possible protective role of HCQ against SARS-CoV-2 cannot be completely dismissed because HCQ may take several weeks to reach its maximal activity [15], and drugs with decreased antimicrobial activity may have some roles as pre-exposure prophylaxis, as shown in the case of doxycycline as prophylaxis against malaria [16].

In this study, to evaluate the possible role of HCQ as pre-exposure prophylaxis against COVID-19, we investigated the prevalence of positive test results for SARS-CoV-2 according to recent HCQ use by using national health-insurance (NHI) data of South Korea.

## 2. Materials and Methods

### 2.1. Study Design and Database

We performed a retrospective matched-cohort study. The source population were individuals who underwent reverse-transcription polymerase chain reaction (RT-PCR) for SARS-CoV-2. Prior HCQ users were defined as “cases” (exposed) and non-HCQ users as “controls” (unexposed). The main outcome was a positive PCR result for SARS-CoV-2 testing. We used the claims database of the South Korean National Health Insurance Service (NHIS) for individuals who underwent a test for SARS-CoV-2 during the study period (1 January 2020–15 May 2020). These claims data were first released by the Ministry of Health and Welfare of South Korea for public purposes on 27 March 2020, and the database was updated on 27 May 2020 to include records through 15 May 2020. The data encompass all claimed healthcare records, including medical visits, prescriptions, procedures, and surgeries. The NHIS provides universal insurance coverage. Healthcare providers are required to claim medical services performed for reimbursement by the government. Given that the data were being claimed for the legitimate interests of healthcare providers, we assumed that few or no values were missing in the database.

In South Korea, testing for the diagnosis of COVID-19 is recommended in the following cases: (i) the presence of fever (37.5 °C or higher) or respiratory symptoms (cough, shortness of breath, etc.) within 14 days of contact with a confirmed COVID-19 patient; (ii) suspected of having COVID-19 according to a physician’s opinion for reasons such as pneumonia of unknown cause; (iii) history of overseas travel within 14 days regardless of symptoms; (iv) epidemiological link to a domestic COVID-19 cluster regardless of symptoms; (v) the presence of fever or respiratory symptoms within 14 days after contact with family or friend who entered South Korea within 14 days; or (vi) history of visit to a place with possible temporal or spatial close contact with a confirmed patient (regardless of symptoms). In the aforementioned cases, the cost of RT-PCR tests is covered by the national health insurance program.

The reimbursement records for individuals over the past three years (1 January 2017–15 May 2020) were collected. COVID-19 diagnosis was validated by linkage with the registry of laboratory-confirmed COVID-19 patients from Korea Disease Control and Prevention (KCDC). We identified individuals aged 18 years or older who were tested for SARS-CoV-2 at least once between 20 January 2020 and 15 May 2020. The prevalence of positive results for SARS-CoV-2 tests in individuals with recent HCQ use was compared with that in individuals without recent HCQ use.

### 2.2. Patient Consent Statement

This study was approved by the Institutional Review Board of Asan Medical Center (S2020-0903). The requirement for written or verbal consent from patients was waived on the basis of the observational nature of the study and the fact that patient identifiers were fully encrypted prior to analysis.

### 2.3. Definitions

We identified individuals who had a reimbursement code (D6584) for the RT-PCR test for the detection of SARS-CoV-2. The date of the first test for SARS-CoV-2 was designated as the index date. Recent use of HCQ was determined when the reimbursement records for drugs confirmed that HCQ was continuously prescribed for at least 30 days until the index date. History of prescription for various drugs, including HCQ and other immunosuppressants (Table S1), was analyzed. Underlying comorbidities were identified using International Classification of Diseases, Tenth Revision (ICD-10) codes when two or more hospital visits with the relevant diagnostic codes within a year prior to the index date were recorded. Death was determined by identifying all inpatient claims records that indicated death. Diagnostic codes and drug codes used in this study are summarized in Table S1.

### 2.4. Statistical Analysis

Categorical variables are presented as frequencies (percentages) and continuous variables are presented as mean (standard deviation). Statistical comparisons were carried out using the Mann–Whitney U, Student’s *t*-, χ^2^, or Fisher’s exact test as appropriate. 

To control for possible selection and indication bias, a propensity score (PS)-matched cohort comprising HCQ users and non-HCQ users was created and adjusted for potential confounders, including age, sex, types of insurance coverage, diabetes, hypertension, ischemic heart disease, heart failure, dyslipidaemia, chronic lung disease, chronic renal disease, chronic liver disease, inflammatory bowel disease, hematologic malignancy, solid tumor, solid-organ transplant, and depression. After calculating the predicted probabilities, each individual in the HCQ group was matched with those in the non-HCQ group at a 1:4 ratio using the propensity scores. Propensity score-matched pairs were created using calipers of width equal to 0.1 of the standard deviation of the logit of the propensity score. We applied greedy nearest-neighbor matching, where each treated unit is sequentially matched with the nearest *k* = 4 control units, without replacement and in the descending order of the propensity score.

We employed the standardized difference of means (SDM) to assess differences in baseline characteristics, and SDM less than 0.1 indicated negligible difference. Model discrimination was assessed with C statistics, and model calibration was assessed with the Hosmer–Lemeshow statistics in the propensity score model to predict the use of HCQ. Odds ratios and corresponding confidence intervals for positive SARS-CoV-2 test results were calculated by conditional logistic regression in the matched samples. 

Since the prevalence of COVID-19 was disproportionately observed between geographic regions due to large-scale outbreaks in specific regions in February 2020 (city of Daegu and Gyeongbuk province), we adjusted the regional variable in the final analysis after propensity-score matching. However, this covariate was not included in the propensity-score model because it was unlikely that the region of residence affected prior HCQ use. In addition, several sensitivity analyses were performed to test the robustness of our findings. This included analyses in which patients were restricted to those who received long-term HCQ treatment for at least 3 or 6 months. Lastly, all analyses were repeatedly performed in patients with rheumatic diseases.

All reported *P* values are two-sided, and those less than 0.05 were considered statistically significant. Data manipulation and statistical analyses were conducted using SAS version 9.4 (SAS Institute Inc., Cary, NC, USA).

## 3. Results

**Figure 1:**
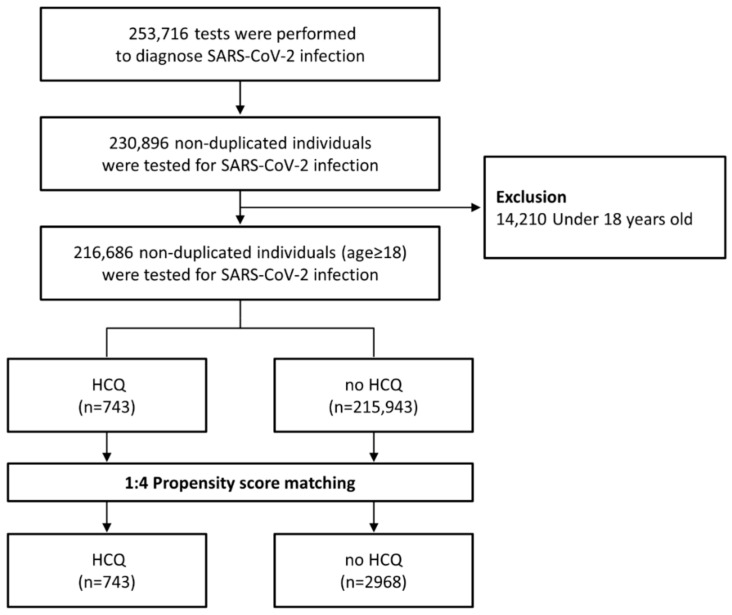
Flowchart of population selection and propensity-score-matched analysis.

A total of 253,716 tests for SARS-CoV-2 were performed during the study period. After excluding cases of individuals aged under 18 years old, 216,686 nonduplicated individuals who had undergone SARS-CoV-2 tests at least once were identified (Figure 1). The baseline characteristics of the study population are summarized in Table 1. Of 216,686 individuals, 5881 patients (2.7%) tested positive for SARS-CoV-2, and 743 patients (0.3%) were HCQ users. Of HCQ users, 695 (93.5%) and 611 (82.2%) were chronic users who received HCQ treatment for at least 90 and 180 days, respectively. The median prescribed daily dose of HCQ was 200 mg (interquartile range, 200–400; range, 100–800).

Of the 216,686 patients who had undertaken the SARS-CoV-2 test, HCQ users were older, had a higher proportion of females, and had a higher prevalence of underlying comorbidities, including rheumatic diseases, than nonusers did (Table 2). The positive rate of SARS-CoV-2 in the HCQ user group (2.2% (16/743)) was not significantly different from that of the nonuser group (2.7% (5865/215,943), *P* = 0.35). The odds ratio (OR) for positive test results for SARS-CoV-2 according to recent HCQ use was 0.79 (95% confidence interval (CI), 0.48–1.30; Table 3).

We matched HCQ users and nonusers at a 1:4 ratio using propensity scores (C statistics = 0.786, *P* = 0.46 by Hosmer–Lemeshow test). After matching, the baseline variables were well-matched between the two groups except for rheumatologic diseases and the use of immunosuppressive agents (Table 2). The logistic-regression model, including rheumatic diseases and immunosuppressant use to predict propensity scores for HCQ use, was not sufficient as per the Hosmer–Lemeshow goodness-of-fit test (*P* < 0.001). In the propensity-score-matched cohort, the rates of positive SARS-CoV-2 tests in HCQ users and nonusers were 2.2% (16/743) and 3.1% (91/2968), respectively (*P* = 0.18). In this cohort, the OR for positive test results for SARS-CoV-2 according to recent HCQ use was 0.69 (95% CI, 0.40–1.19; Table 3). Moreover, recent HCQ use did not show significant associations with positive test results in multivariate analysis after adjusting for the geographic region where the SARS-CoV-2 tests had been performed (OR, 0.80; 95% CI, 0.43–1.52; *P* = 0.50), or immunosuppressant use (OR, 0.69; 95% CI, 0.34–1.38; *P* = 0.29; Table 3). In addition, results were robust, as per sensitivity analyses in which patients were restricted to those who had received long-term HCQ treatment for more than 3 or 6 months (Table 3). Among patients with COVID-19, all-cause mortality in the HCQ user group (0% (0/16)) did not significantly differ from that in the nonuser group (2.4% (140/5868), *P* > 0.99; Table 2).

The effect of HCQ was further analyzed in subjects restricted to patients with rheumatic disease due to a residual imbalance in covariate of rheumatic disease between HCQ users and nonusers in primary analysis. Among patients with rheumatic disease, the rates of positive SARS-CoV-2 tests were not significantly different between HCQ users and nonusers before and after propensity matching (Table 4). No significant difference was observed after adjusting geographic region or immunosuppressant use in the likelihood of positive test for SARS-CoV-2 according to treatment with HCQ (Table 4). These results were robust in further sensitivity analyses with long-term HCQ users (Table 4).

## 4. Discussion

In this study, using data from subjects who had undergone SARS-CoV-2 testing, we found that the positive rate for SARS-CoV-2 in recent HCQ users was not significantly different from that in non-HCQ users after the adjustment of indication bias by propensity-score matching. These findings were consistently observed in long-term HCQ users treated for more than 3 or 6 months. However, the possible prophylactic role of HCQ against COVID-19 cannot be completely dismissed considering the wide CIs; therefore, cautious interpretation is needed until further randomized clinical trials provide more definite answers. 

Aminoquinolines, including chloroquine and HCQ, showed antiviral effects against coronavirus in in vitro studies [7,8,9]. However, the use of HCQ failed to show significant effects against SARS-CoV-2 infection in randomized clinical trials for the treatment of COVID-19 or for use as postexposure prophylaxis [13,14]. However, more than half of the participants in the postexposure prophylaxis study were enrolled 3 or 4 days after the exposure [14]. Therefore, many patients might pass the incubation period and did not obtain the prophylaxis effect of HCQ. Furthermore, the potential role of HCQ in protecting individuals against COVID-19 was continuously suggested due to its complex pharmacokinetics, including a long half-life and a large volume of distribution [15]. In addition, the weak antimicrobial effect of a drug may exert beneficial roles as pre-exposure prophylaxis, as in the case of doxycycline, which was used as pre-exposure prophylaxis against malaria despite its limited role in malaria treatment [16].

There are limited data on the effectiveness of HCQ use as pre-exposure prophylaxis, but several cases of COVID-19 were reported in patients with a long-term history of HCQ treatment [17,18,19]. In two recent randomized clinical trials conducted on healthcare workers, the incidence of SARS-CoV-2 infection was not significantly lower in those who received HCQ for pre-exposure prophylaxis compared to placebo for 8 or 12 weeks of HCQ use [20,21]. Considering the pharmacokinetics of HCQ, such as long half-life and wide volume of distribution, 8 or 12 weeks may not be sufficient to prevent SARS-CoV-2 infection in healthcare workers at high risk of exposure. However, the prevalence of COVID-19 in patients receiving HCQ for at least 30 days did not significantly differ from that of nonusers in our study. In addition, results were robust in sensitivity analyses with long-term users who had received HCQ for more than 3 or 6 months. Taken together, our findings may support recent randomized clinical trials opposing the administration of HCQ for pre-exposure prophylaxis [20,21]. Given the potential risk of complications, including retinopathy and QT interval prolongation with subsequent arrhythmia following HCQ use, clinicians should carefully weigh the benefits of using this drug [22,23,24,25].

There are several limitations to our study. First, although more than 200,000 nonduplicated cases with SARS-CoV-2 tests were included, the statistical interpretation was limited due to the small numbers of HCQ users and positive SARS-CoV-2 test results. We also cannot rule out the issue of insufficient power to achieve statistical significance in our study due to the low prevalence of positive SAR-CoV-2 PCR during the study period. Second, we were unable to assess the indication of the SARS-CoV-2 test and the degree of exposure to the confirmed cases of each individual. This study was conducted in South Korea, which is a country with wide surveillance for COVID-19, and where testing for SARS-CoV-2 is recommended for any cases of close contact to confirmed COVID-19 patients, regardless of symptoms or signs of COVID-19 [26]. Third, a limitation of the propensity-score-matched method is that it adjusts only for observed covariables. Hence, we cannot rule out the possibility that an unmeasured confounding factor affected our results. In addition, rheumatic disease and immunosuppressants were not included in propensity-score matching because the regression model for predicting the probability of HCQ use did not fit the observed data when these covariates were included. Nevertheless, propensity-score-matched analysis is a reliable method that adjusts treatment indication biases, and is especially useful in cases such as pandemics in which randomization is not feasible or too slow to quickly provide answers to important policy-related questions. In addition, we analyzed the impact of HCQ among patients with rheumatic diseases with adjusting immunosuppressant use, but no significant difference was observed in the rates of positivity for SAR-CoV-2 tests according to HCQ use. Fourth, only the results of SARS-CoV-2 tests and demographic data with disease codes and prescribed drugs were available in the database used in this study. Therefore, we were not able to assess the clinical impact of HCQ use in COVID-19 patients or the likelihood of positive test results according to the compliance of those taking prescribed HCQ.

In conclusion, recent exposure to HCQ was not significantly associated with a lower risk of SARS-CoV-2 infection. Therefore, our data do not support the beneficial role of HCQ against COVID-19 as pre-exposure prophylaxis. The findings of our study should be interpreted with caution due to the above-mentioned limitations. Nevertheless, these data provide several implications to the decision makers in public-health and clinical fields. Despite the lack of statistical significance, difference in positive rates of SARS-CoV-2 tests between HCQ users and nonusers may provide a temporary parameter for calculating sample sizes for use in future clinical trials. Further well-designed clinical trials are needed to provide definite answers on the role of HCQ as pre-exposure prophylaxis.

## Figures and Tables

**Table 1:** Baseline characteristics of patients tested for SARS-CoV-2.

	n (%)
	Patients Tested for SARS-CoV-2
Characteristics	(n = 216,686)
Age, mean (SD), y	49.4 (19.9)
<50	115,759 (53.4)
≥50	100,927 (46.6)
Sex	
Male	102,754 (47.4)
Female	113,932 (52.6)
Insurance type	
National health insurance	204,421 (94.3)
Medicaid and veteran health care	12,265 (5.7)
Region ^a^	
Seoul metropolitan area	108,570 (50.1)
Daegu–Gyeongbuk	34,309 (15.8)
Other	73,807 (34.1)
Underlying diseases	
Hypertension	60,441 (27.9)
Dyslipidemia	59,735 (27.6)
Chronic lung diseases	49,203 (22.7)
Diabetes	35,636 (16.4)
Solid tumor	22,571 (10.4)
Ischemic heart disease	15,290 (7.1)
Heart failure	13,086 (6)
Chronic kidney disease	9665 (4.5)
Liver cirrhosis	3144 (1.5)
Depression	2511 (1.2)
Inflammatory bowel disease	685 (0.3)
Hematologic malignancy	1552 (0.7)
Solid-organ transplantation	1220 (0.6)
Rheumatic diseases	
Systemic lupus erythematosus	465 (0.2)
Rheumatoid arthritis	4152 (1.9)
Spondyloarthropathy	526 (0.2)
Immunosuppressants use	
Biologics	43 (0.02)
Corticosteroid	8478 (3.9)
Other	2656 (1.2)
Outcome	
Positive test result for SARS-CoV-2	5881 (2.7)
Mortality due to COVID-19	140/5881 (2.3)

Abbreviations: SARS-CoV-2, severe acute respiratory syndrome coronavirus 2; SD, standard deviation. ^a^ Region indicates where SARS-CoV-2 test was performed.

**Table 2:** Baseline demographic characteristics, prevalence of positive test results, and COVID-19 mortality of patients tested for SARS-CoV-2 according to hydroxychloroquine (HCQ) use.

	Before Matching, n (%)				After 1:4 Matching, n (%)			
Characteristics	HCQ Users (n = 743)	Nonusers (n = 215,943)	*P*	SDM	HCQ Users (n = 743)	Nonusers (n = 2968)	*P*	SDM
Age, mean (SD), y	56.6 (17.1)	49.4 (19.9)	<0.001	0.388	56.6 (17.1)	57.3 (17.4)	0.35	−0.039
<50	250 (33.6)	115,509 (53.5)	<0.001	−0.408	250 (33.6)	970 (32.7)	0.62	0.021
≥50	493 (66.4)	100,434 (46.5)			493 (66.4)	1998 (67.3)		
Sex								
Male	134 (18.0)	102,620 (47.5)	<0.001	−0.662	134 (18.0)	536 (18.1)	>0.99	−0.001
Female	609 (82.0)	113,323 (52.5)			609 (82.0)	2432 (81.9)		
Insurance type								
National health insurance	677 (91.1)	203,744 (94.4)	<0.001	−0.125	677 (91.1)	2723 (91.7)	0.58	−0.022
Medicaid and veteran healthcare	66 (8.9)	12,199 (5.6)			66 (8.9)	245 (8.3)		
Underlying diseases								
Hypertension	340 (45.8)	60,101 (27.8)	<0.001	0.378	340 (45.8)	1312 (44.2)	0.45	0.031
Dyslipidemia	410 (55.2)	59,325 (27.5)	<0.001	0.586	410 (55.2)	1709 (57.6)	0.24	−0.048
Chronic lung diseases	276 (37.1)	48,927 (22.7)	<0.001	0.321	276 (37.1)	1139 (38.4)	0.54	−0.025
Diabetes	165 (22.2)	35,471 (16.4)	<0.001	0.147	165 (22.2)	660 (22.2)	>0.99	−0.001
Solid tumor	88 (11.8)	22,483 (10.4)	0.20	0.046	88 (11.8)	382 (12.9)	0.45	−0.031
Ischemic heart disease	81 (10.9)	15,209 (7)	<0.001	0.135	81 (10.9)	295 (9.9)	0.44	0.032
Heart failure	84 (11.3)	13,002 (6)	<0.001	0.189	84 (11.3)	292 (9.8)	0.24	0.048
Chronic kidney disease	75 (10.1)	9590 (4.4)	<0.001	0.219	75 (10.1)	243 (8.2)	0.10	0.066
Liver cirrhosis	19 (2.6)	3125 (1.4)	0.01	0.079	19 (2.6)	50 (1.7)	0.12	0.061
Depression	14 (1.9)	2497 (1.2)	0.06	0.06	14 (1.9)	53 (1.8)	0.86	0.007
Inflammatory bowel disease	2 (0.3)	683 (0.3)	0.82	−0.009	2 (0.3)	5 (0.2)	0.57	0.022
Hematologic malignancy	11 (1.5)	1541 (0.7)	0.01	0.074	11 (1.5)	41 (1.4)	0.84	0.008
Solid-organ transplantation	7 (0.9)	1213 (0.6)	0.17	0.044	7 (0.9)	17 (0.6)	0.30	0.043
Rheumatic diseases								
Systemic lupus erythematosus	231 (31.1)	234 (0.1)	<0.001	0.944	231 (31.1)	12 (0.4)	<0.001	0.929
Rheumatoid arthritis	533 (71.7)	3619 (1.7)	<0.001	2.116	533 (71.7)	107 (3.6)	<0.001	1.977
Spondyloarthropathy	6 (0.8)	520 (0.2)	0.002	0.079	6 (0.8)	10 (0.3)	0.11	0.062
Immunosuppressant use								
Biologics	1 (0.1)	42 (0)	0.14	0.042	1 (0.1)	0 (0)	0.20	0.052
Corticosteroid	447 (60.2)	8031 (3.7)	<0.001	1.521	447 (60.2)	166 (5.6)	<0.001	1.427
Other	294 (39.6)	2362 (1.1)	<0.001	1.088	294 (39.6)	50 (1.7)	<0.001	1.060
Outcome								
Positive test result for SARS-CoV-2	16 (2.2)	5865 (2.7)	0.35		16 (2.2)	91 (3.1)	0.18	
Mortality due to COVID-19	0/16 (0)	140/5865 (2.4)	>0.99		0/16 (0)	0/91 (0)	>0.99	

Abbreviations: HCQ, hydroxychloroquine; SDM, standardized difference in means.

**Table 3:** Likelihood of positive test for SARS-CoV-2 according to treatment with HCQ.

	Odds Ratio	95% CI	*P*
**Primary Analysis (HCQ ≥ 1 Month)**			
Unadjusted analysis	0.79	0.48–1.30	0.35
PS-matching cohort			
unadjusted	0.69	0.40–1.19	0.18
Adjusted for region ^a^	0.80	0.43–1.52	0.50
Adjusted for immunosuppressant use ^b^	0.69	0.34–1.38	0.29
**Sensitivity Analyses**			
Unadjusted analysis			
HCQ ≥ 3 months	0.74	0.43–1.25	0.26
HCQ ≥ 6 months	0.78	0.45–1.35	0.37
PS-matching cohort			
HCQ ≥ 3 months	0.69	0.39–1.22	0.20
HCQ ≥ 6 months	0.59	0.32–1.07	0.08

Abbreviations: CI, confidence interval; PS, propensity score. ^a^ Region indicates where the test for SARS-CoV-2 was performed. ^b^ Immunosuppressant use was defined as prescription of corticosteroids, biologics, or other medications summarized in Table S1.

**Table 4:** Likelihood of positive test for SARS-CoV-2 according to treatment with HCQ among patients with rheumatic diseases.

	Odds Ratio	95% CI	*P*
**Primary Analysis (HCQ ≥ 1 Month)**			
Unadjusted analysis	0.93	0.53–1.63	0.79
PS matching cohort			
unadjusted	0.85	0.48–1.53	0.59
Adjusted for region ^a^	1.07	0.55–2.07	0.85
Adjusted for immunosuppressant use ^b^	0.84	0.45–1.54	0.57
**Sensitivity Analyses**			
HCQ ≥ 3 months	0.85	0.46–1.54	0.58
HCQ ≥ 6 months	0.88	0.47–1.64	0.68

Abbreviations: CI, confidence interval; PS, propensity score. ^a^ Region indicates where the test for SARS-CoV-2 was performed. ^b^ Immunosuppressants use was defined as the prescription of corticosteroids, biologics, or other medications summarized in Table S1.

## Data Availability

The data underlying this article were provided by participating in the Global Research Collaboration Project on COVID-19, jointly hosted by the Ministry of Health and Welfare of Korea and the Health Insurance Review and Assessment Service of Korea. This project was launched on 27 March 2020 in response to the COVID-19 pandemic, and was scheduled to end on 31 July 2020. The notice for collaboration project closing was announced in the following URL: https://hira-covid19.net/view_community?community_id=1595897139705 (accessed on 1 January 2021).

## Supplementary Materials

The following are available online at https://www.mdpi.com/1999-4915/13/2/329/s1, Table S1. Diagnostic codes and drug codes used in this study.
