# Supplementary material for: Recent Hydroxychloroquine Use Is Not Significantly Associated with Positive PCR Results for SARS-CoV-2: A Nationwide Observational Study in South Korea
Source: Viruses. 2021 Feb 20;13(2):329. doi: 10.3390/v13020329 (PMC7924588; doi:10.3390/v13020329)
Supplement: Supplementary file 1 [file viruses-13-00329-s001.pdf]

Supplementary data legends

Table S1. Diagnostic codes and drug codes used in this study.

Table S1. Diagnostic codes and drug codes used in this study.

**Diagnoses**		
**Underlying diseases**[a]		
	Hypertension	I10, I11, I12, I13, I15
	Dyslipidemia	E78
	Chronic lung diseases	J40, J41, J42, J43, J44, J45, J46, J47, J60, J61, J62, J63, J64, J65, J66, J67, I278, I279, J684, J701, J703
	Diabetes	E10, E11, E12, E13, E14
	Solid tumor	C0, C1, C2, C3, C4, C5, C6, C7, C80, C97
	Ischemic heart disease	I20, I21, I22, I23, I24, I25
	Heart failure	I50, I43, I099, I110, I130, I132, I255, I420, I425, I426, I427, I428, I429
	Chronic kidney disease	N03, N04, N18, N19, N25, N26, Z49, Z94, I120, I131, Z992
	Liver cirrhosis	K72, I850, I859, I864, I980, I983, K703, K704, K711, K715, K743, K744, K745, K746, K754, K762, K765, K766, K767
	Depression	F34, F322, F323, F332, F333
	Inflammatory bowel disease	K50, K51
	Hematologic malignancy	C81, C82, C83, C84, C85, C86, C87, C88, C89, C90, C91, C92, C93, C94, C95, C96
	Solid organ transplantation	Z940, Z941, Z942, Z943, Z944, Z948
	Rheumatic diseases	
	Systemic lupus erythematosus	M05, M06
	Rheumatoid arthritis	L93, M32
	Spondyloarthropathy	M45
**Medications**[b]		
**Main exposure**	hydroxychloroquine	171701ATB, 171702ATB, 171704ATB, 171705ATB, 171703ATB
**Immunosuppressants**		
	Biologics	
	abatacept	512201BIJ, 512230BIJ
	adalimumab	488401BIJ, 488430BIJ, 488431BIJ, 488433BIJ
	anakinra	517801BIJ
	etanercept	455801BIJ, 455802BIJ, 455803BIJ, 455830BIJ, 455831BIJ
	golimumab	621201BIJ, 621202BIJ, 621203BIJ, 621230BIJ, 621231BIJ, 621232BIJ
	infliximab	383501BIJ
	leflunomide	
	rituximab	422601BIJ, 422602BIJ, 422603BIJ, 422630BIJ, 422631BIJ, 422632BIJ
	tocilizumab	520401BIJ, 520402BIJ, 520403BIJ, 520404BIJ, 520430BIJ, 520431BIJ, 520432BIJ, 520433BIJ
	tofacitinib	627201ATB, 627202ATB
	baricitinib	667901ATB, 667902ATB
	Corticosteroids	
	prednisolone	217001ATB, 217003ASY, 217004ASY, 217030ASY, 217034ASY, 217035ASY, 217302BIJ
	methylprednisolone	193302ATB, 193305ATB, 193501BIJ, 193502BIJ, 193530BIJ, 193531BIJ, 193601BIJ, 193602BIJ, 193603BIJ, 193604BIJ
	hydrocortisone	170901ATB, 170905ATB, 170906ATB, 171201BIJ, 171202BIJ

	dexamethasone	141901ATB, 141903ATB, 141904ATB, 142001BIJ, 142030BIJ, 142201BIJ, 142202BIJ, 142230BIJ, 142232BIJ, 142233BIJ
	triamcinolone	243201ATB, 243202ATB, 243203ATB, 243301BIJ, 243303BIJ, 243305BIJ, 243335BIJ, 243336BIJ, 243337BIJ
	deflazacort	140801ATB
	Other immunosuppressants	
	methotrexate	192101ATB, 192107ATB, 192102BIJ, 192103BIJ, 192104BIJ, 192105BIJ, 192107BIJ, 192108BIJ, 192109BIJ, 192110BIJ, 192111BIJ, 192132BIJ, 192134BIJ, 192136BIJ, 192138BIJ, 192139BIJ, 192140BIJ, 192141BIJ, 192142BIJ, 192143BIJ, 192144BIJ
	leflunomide	434601ATB, 434602ATB
	ciclosporin (cyclosporin A)	139201ACS, 139201ACS, 139204ACS, 139202BIJ, 139230BIJ, 194701ACS, 194701ALQ, 194702ACS, 194730ALQ, 194731ALQ
	cyclophosphamide	139001ATB, 139003BIJ, 139004BIJ, 139005BIJ
	tacrolimus	234201ACH, 234201ACH, 234201ATB, 234202BIJ, 234203ACH, 234203ATB, 234204ACH, 234204ATB, 234205ACR, 234206ACR, 234207ACR, 234208ATB, 234230BIJ
	sirolimus	485501ATB, 485502ATB, 485503ATB
	azathioprine	112401ATB, 112402ATB
	sulfasalazine	232801ATE
	mycophenolate mofetil	197801ACH, 197802ATB, 197804ASS, 197830ASS, 451401ATE, 451402ATE
	everolimus	485601ATB, 485602ATB, 485603ATB, 485604ATB, 485605ATB, 485606ATB, 485607ATB
	bucillamine	348201ATB
	mizoribine	196601ATB, 196602ATB
**Outcome**[c]		
	Laboratory confirmed COVID-19	RAW.co19_t200_trans_dn WHERE CONFIRM IN ('Y');
	Death	RAW.co19_t200_trans_dn WHERE DEATH IN ('Y');

[a] Based on the International Classification of Disease, 10th Revision codes.

[b] Based on World Health Organization-Anatomical Therapeutic Chemical classification codes.

[c] The diagnosis of COVID-19 and outcome of death identified by SAS codes which were validated by linkage with the registry of laboratory-confirmed COVID-19 patients from the Korea Disease Control and Prevention (KCDC).
